# Reimagining Engagement and Connection: Usability Insights From Virtual Reality Use Among Older Adults

**DOI:** 10.1093/geroni/igaf122.4311

**Published:** 2025-12-31

**Authors:** Pallabi Bhowmick, Noah Olivero, Tracy Mitzner, Avinash Gupta, Wendy Rogers

**Affiliations:** University of Illinois Urbana-Champaign, Champaign, Illinois, United States; University of Illinois Urbana-Champaign, Champaign, Illinois, United States; Person in Design, Atlanta, Georgia, United States; University of Illinois Urbana-Champaign, Champaign, Illinois, United States; University of Illinois Urbana-Champaign, Champaign, Illinois, United States

## Abstract

Social isolation affects nearly one in four older adults, and virtual reality (VR) has emerged as a promising tool for fostering social connection and engagement. To better understand its potential, we have conducted a needs assessment study with older adults to explore the usability, acceptance, and perceived benefits of VR applications across multiple sites. Analysis of the data from one site (n = 16, aged 65–79, M = 71.56, SD = 4.18) showed that majority of the participants (88%) rated enjoyability, engagement, and appeal between 7 and 10 on a 10-point scale, indicating strong enthusiasm for VR as a novel technology offering a fun alternative to support cognitive engagement and leisure activities. Additionally, participants emphasized the social potential of VR and its ability to facilitate meaningful connection with distant family members, particularly grandchildren, through gaming and collaborative activities. They expressed excitement about experiences that transcend physical limitations, such as virtual travel, noting that immersive VR offers a compelling alternative for exploring new places with friends and family as travel becomes difficult with age. Participants also preferred interactive over passive experiences, highlighting a desire for activities that enabled active engagement within the VR environment. Although some participants initially found the VR applications challenging due to unfamiliarity, they emphasized the need for practice rather than additional training. Many expressed a willingness to adapt and highlighted the importance of home access to support continued use. These findings challenge ageist assumptions about older adults and technology, underscoring the need for inclusive VR design that enhances engagement and quality of life.

